# Medical Items

**Published:** 1874-05

**Authors:** 


### Medical Items.

Dr. B. F. Dawson announces in the last number of the *American Journal of Obstetrics* his withdrawal from the editorial management of that journal. Dr. Paul F. Munde succeeds him. Under the management of Dr. Dawson the Journal has gained a wide reputation which will no doubt be fully sustained by his able successor. Dr. Dawson announces that he has founded an annual prize of $150 in gold for the best essay on some subject to be announced at the beginning of each year in the Journal. The subject for this year is "Congenital Deformities and Diseases Depending on Maladies of the Uterus or Membranes." The competing essays must be sent to the office of the publishers of the Journal, (Messrs. Wm. Wood & Co., New York City,) on or before April 15, 1875. Each essay should be accompanied by the name of the author in a sealed envelope.

The Alumni of the Albany Medical College have formed an association and adopted a constitution and by-laws, a copy of which we have received. Dr. H. D. Didama, of Syracuse, is President, Dr. W. G. Tucker, of Albany, Secretary, and Dr. Jas. S. Bailey Treasurer. Any graduate of the Albany Medical College in good standing, can become a member of the association by forwarding the annual fee, one dollar, to the Treasurer, Dr. J. S. Baily, 95 Eagle St., Albany, N. Y.——At the last commencement of the Philadelphia College of Pharmacy, the degree of G. P. was conferred on eighty one graduates. We notice as number three on the list the name of Mr. Alexander King, of this city now with Wm. King, Jr., Druggist.——The editor of the *New Orleans Medical and Surgical Journal* accuses us of being uncharitable n that we had withdrawn our sympathy from the poor South Carolina doctor who cannot associate with the Northern Section of the American Medical Association. He asks, and justly, too, "Who is able to say that some irreparable loss or incurable grief has not driven this physician to assume a position which no one seeks to justify ?" We certainly cannot, and would not by our words add a feather's weight to his sorrow ; yet we cannot feel that sympathy for a man which we should who brings the remembrance of past political defeats into his relation with medical or scientific associations. The editor is certainly wrong in one fact, we do have a large amount of sympathy for the defeated, and we fear, badly governed people of the South, but our feeling of charity and kindness cannot help being lessened by such expressions as those contained in the letter alluded to, which we did not, however, suppose to represent the sentiments of the whole people. Does the Editor suppose that if a Northern Surgeon should be invited to be present at the approaching meeting of ex-Confederate Surgeons at Atlanta, which in its scientific character, although reviving old memories, has nothing to do with political feelings, he would refuse, even though his friends and brothers had fallen on southern fields or starved in Andersonville and Libby.—*The Missour Clinical Resord* is the title of a new Medical Monthly published by the Faculy of the Missouri Medical College. It is under the Editorial supervision of Drs. HARDAWAY, SHAW and TODD——Messrs. WM. WOOD & Co. announce that they will publish by subscription a translation of an Encyclopedia of the Practice of Medicine, shortly to appear in Germany. This work will be issued in fifteen volumes, at the rate of three or four per year. From the prospectus which we have seen we infer that the German edition will be under the supervision of Prof. H. Von Ziemssen, of Erlangen ; the various subjects are to be written upon by some of the most prominent German physicians. The work promises to be a valuable hand-book of practical and scientific medicine. The translation of the American edition is to be undertaken by thoroughly competent physi[illegible]ians.——From present indication it is probable that the American Medical Association, at Detroit, will be largely attended. We are unable to say whether any steps will be taken to make the needed changes in the Association which have been so much discussed, or not, but presume that steps in that direction will be inaugurated.——The Association of American Medical Editors meets Monday evening, June 1st, in Detroit ; it is hoped the meeting will be fully attendad.

